# Supplementary figures and images for: Causal Relationship between Meat Intake and Biological Aging: Evidence from Mendelian Randomization Analysis
Source: Nutrients. 2024 Jul 26;16(15):2433. doi: 10.3390/nu16152433 (PMC11313912; doi:10.3390/nu16152433)

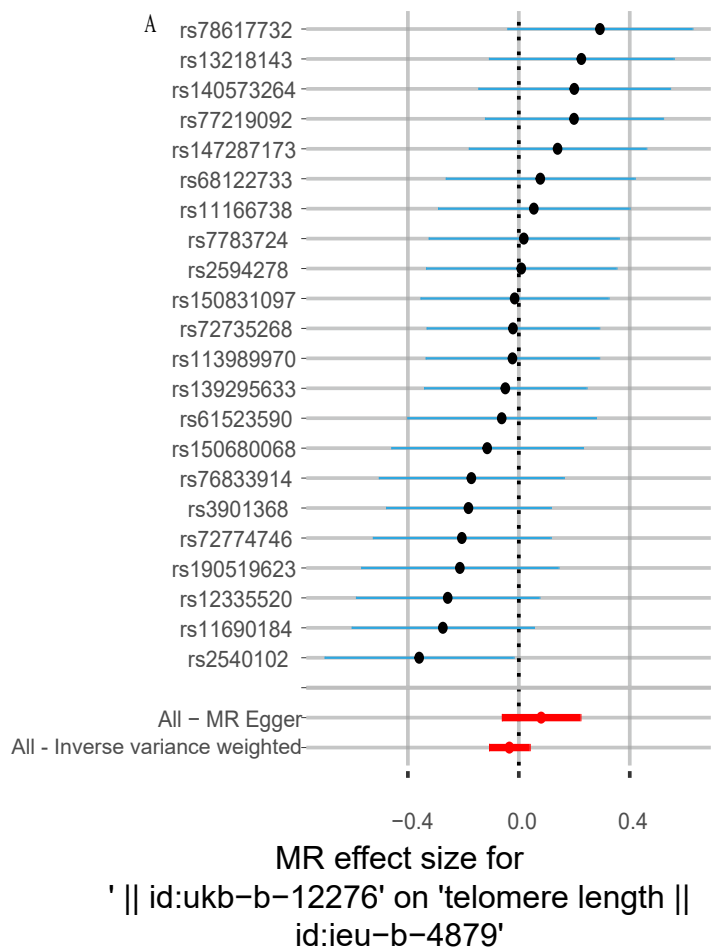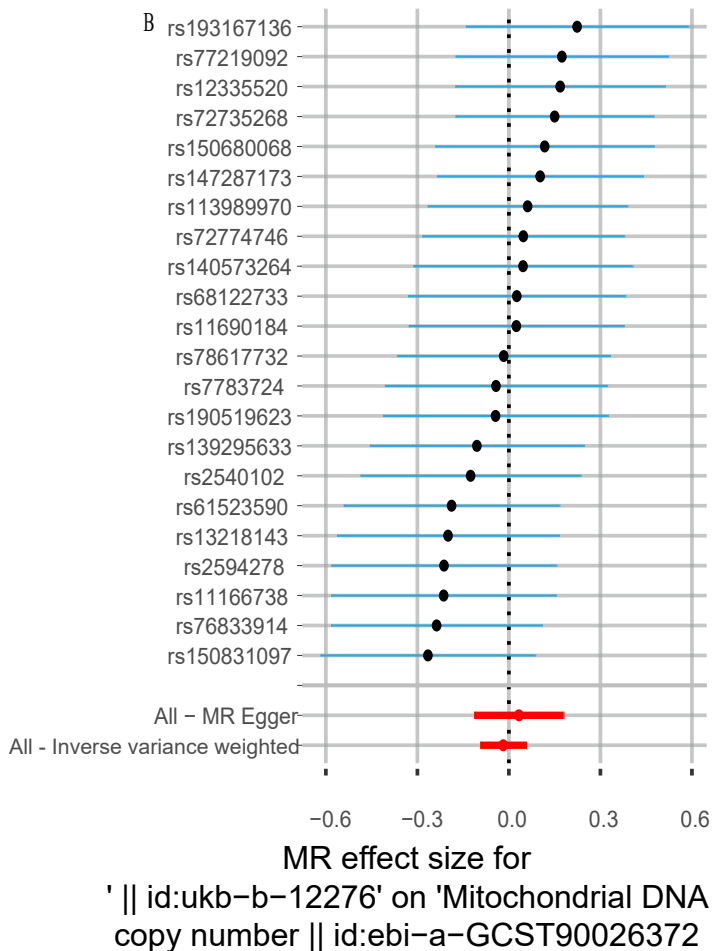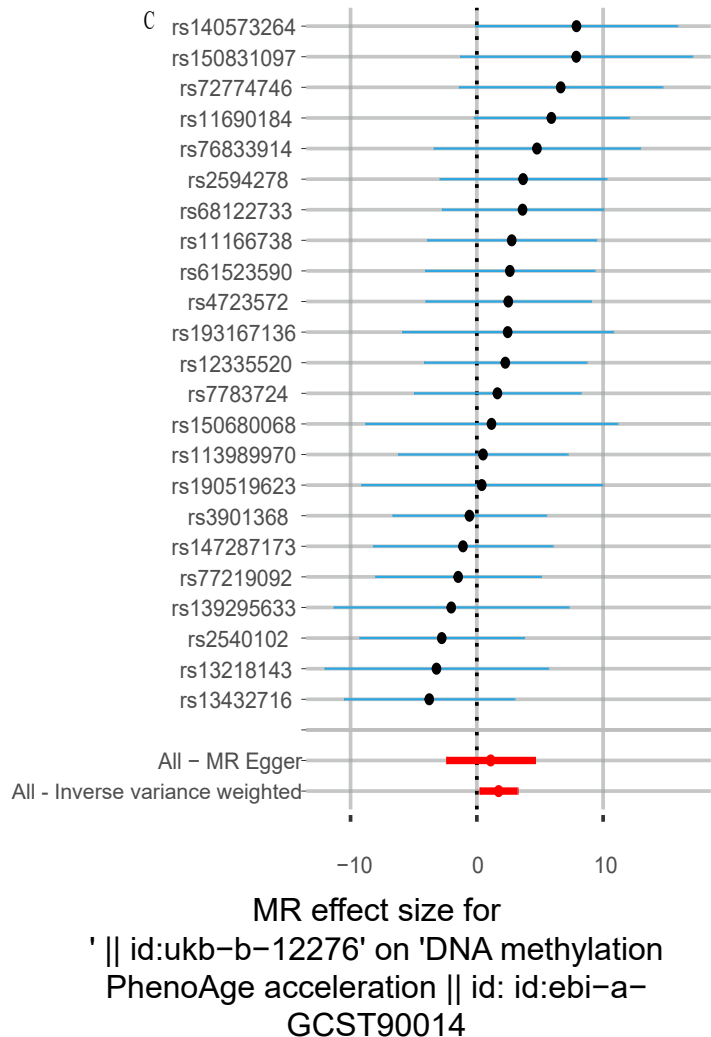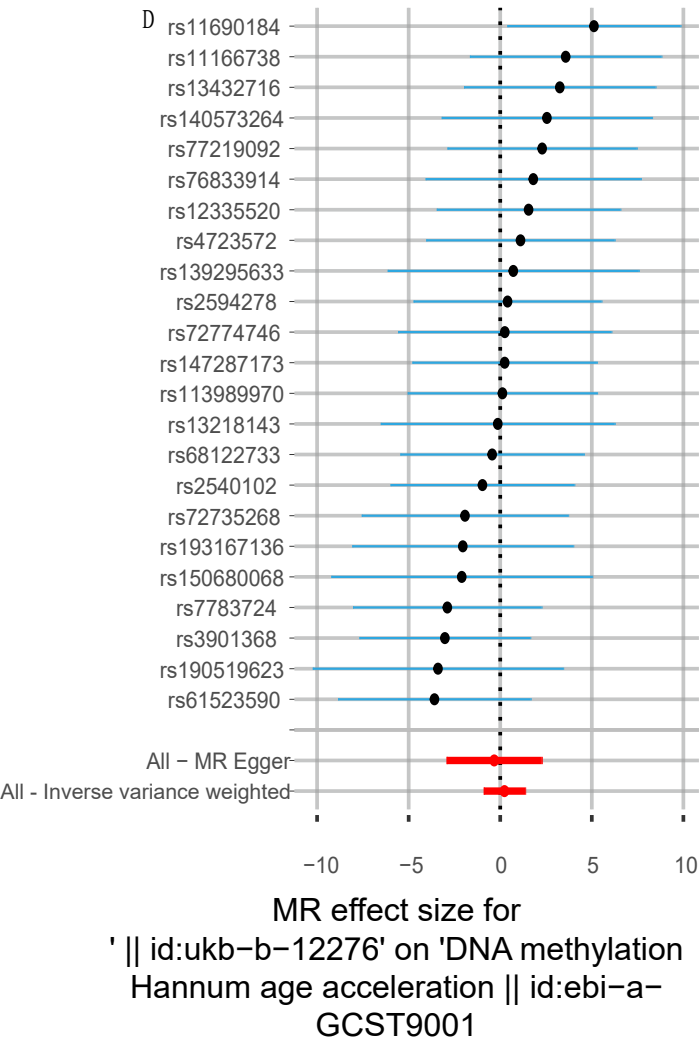

Supplement: Supplementary file 1 [file nutrients-16-02433-s001.zip › Supplementary materials/Figure S1 The causal effect for single SNP for the MR analysis between meat consumers and aging phenotypes.pdf]

MR Method

Inverse variance weighted MR Egger

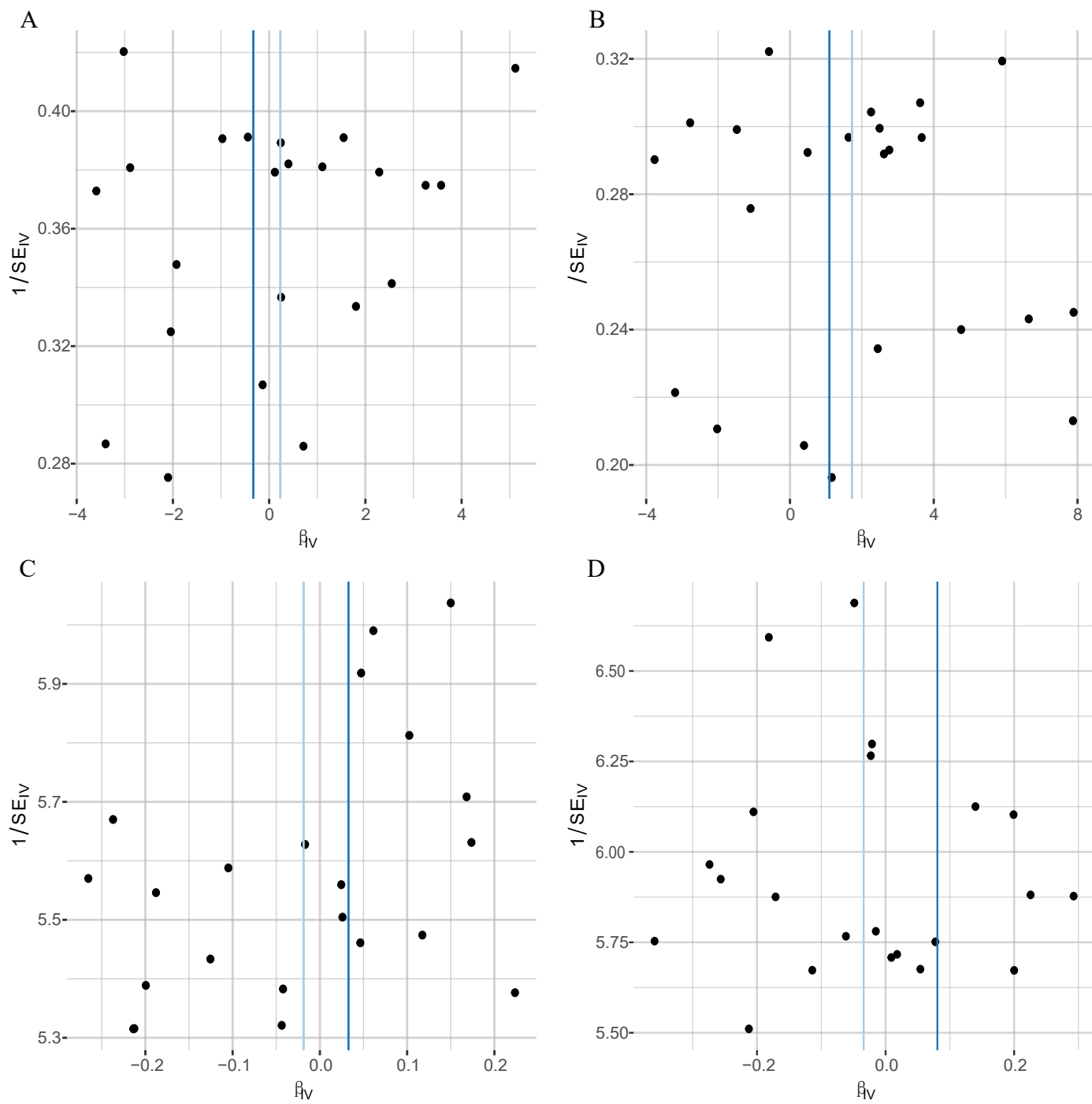

Supplement: Supplementary file 1 [file nutrients-16-02433-s001.zip › Supplementary materials/Figure S2 Funnel plots for the MR analysis between meat consumers and aging phenotypes.pdf]

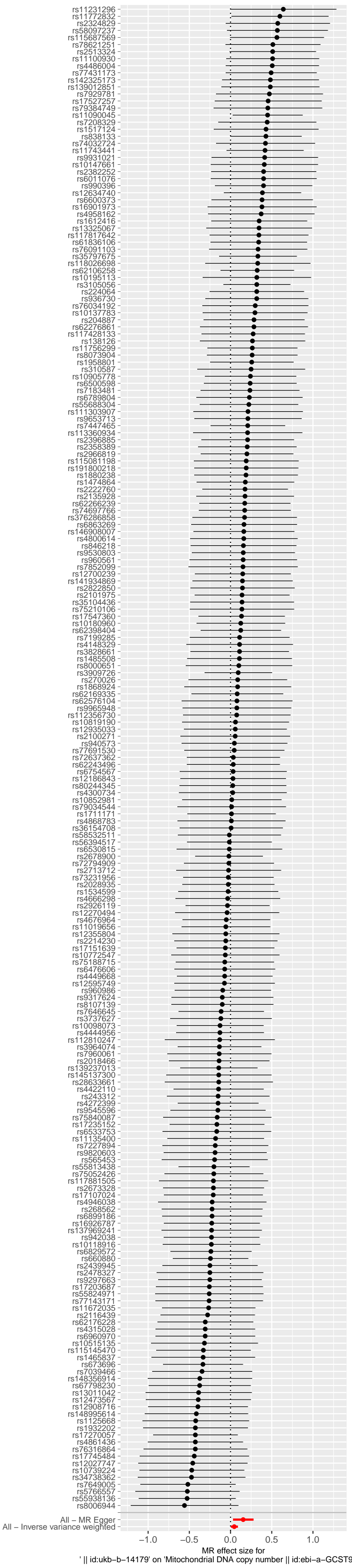

Supplement: Supplementary file 1 [file nutrients-16-02433-s001.zip › Supplementary materials/Figure S3 Single SNP forest plot of MR analysis between lamb intake and mtDNA copy number.pdf]

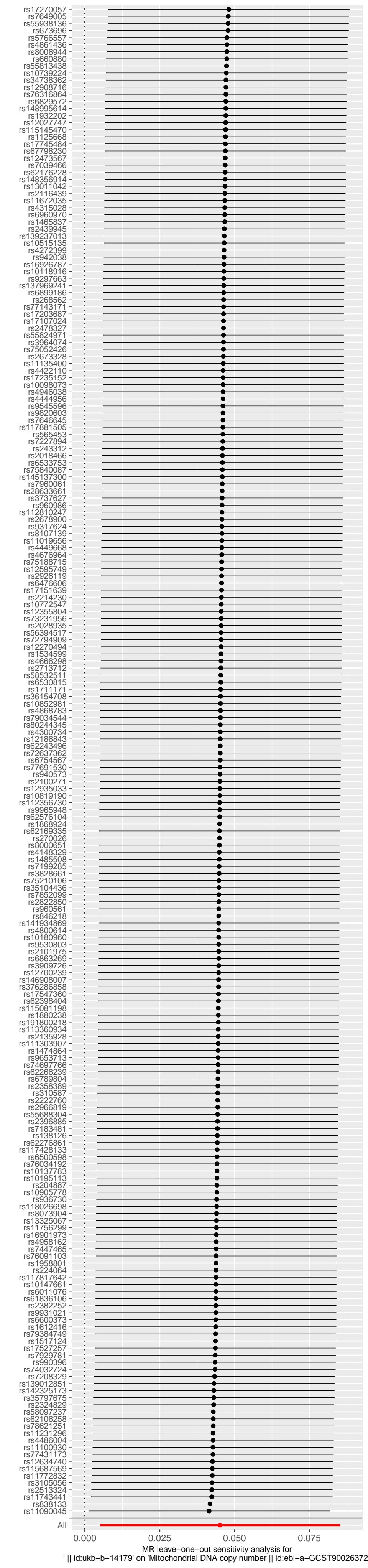

Supplement: Supplementary file 1 [file nutrients-16-02433-s001.zip › Supplementary materials/Figure S4 Leave-one-out plot of MR analysis between lamb intake and mtDNA copy number.pdf]

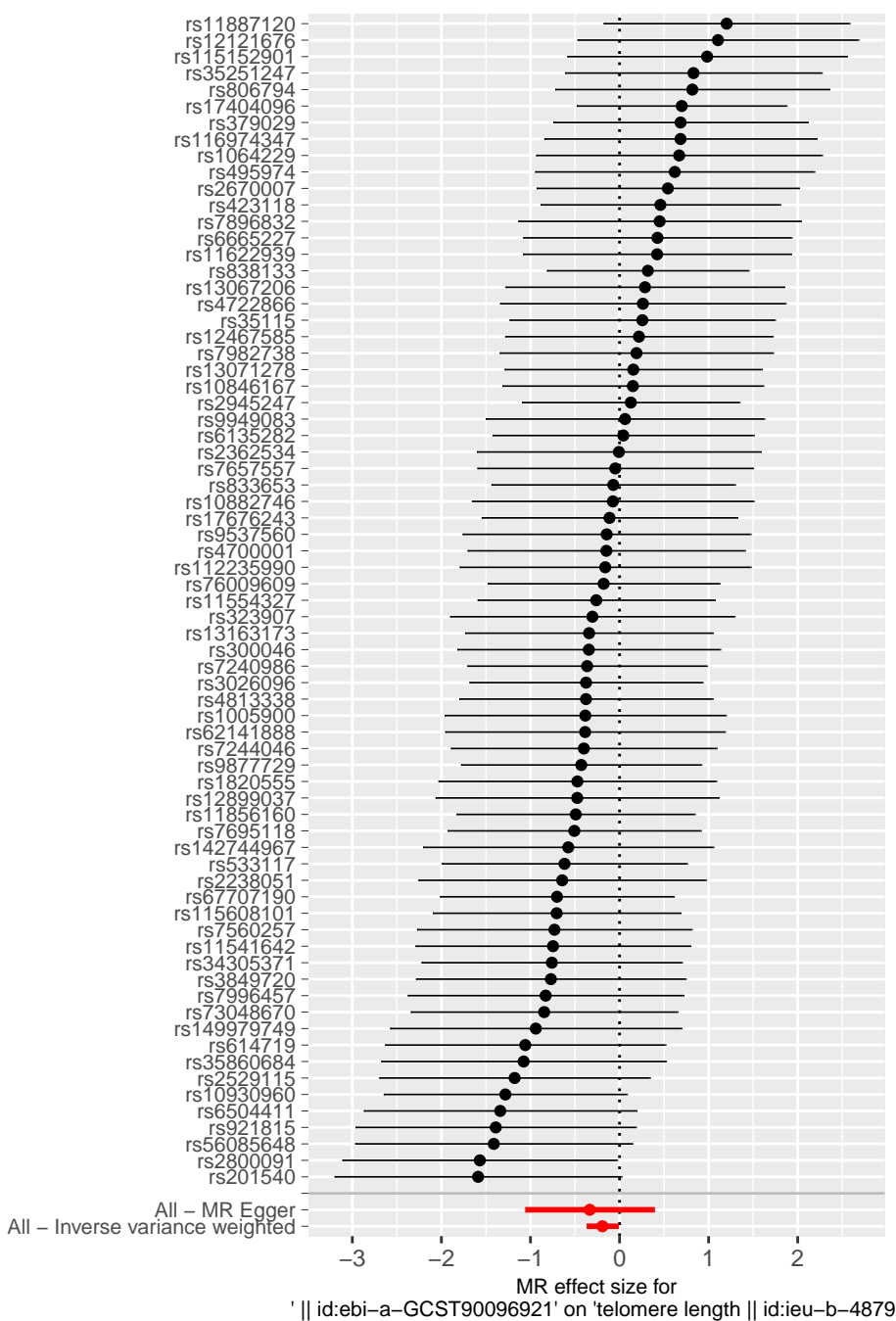

Supplement: Supplementary file 1 [file nutrients-16-02433-s001.zip › Supplementary materials/Figure S5 Single SNP forest plot of MR analysis between processed meat intake and telonere length.pdf]

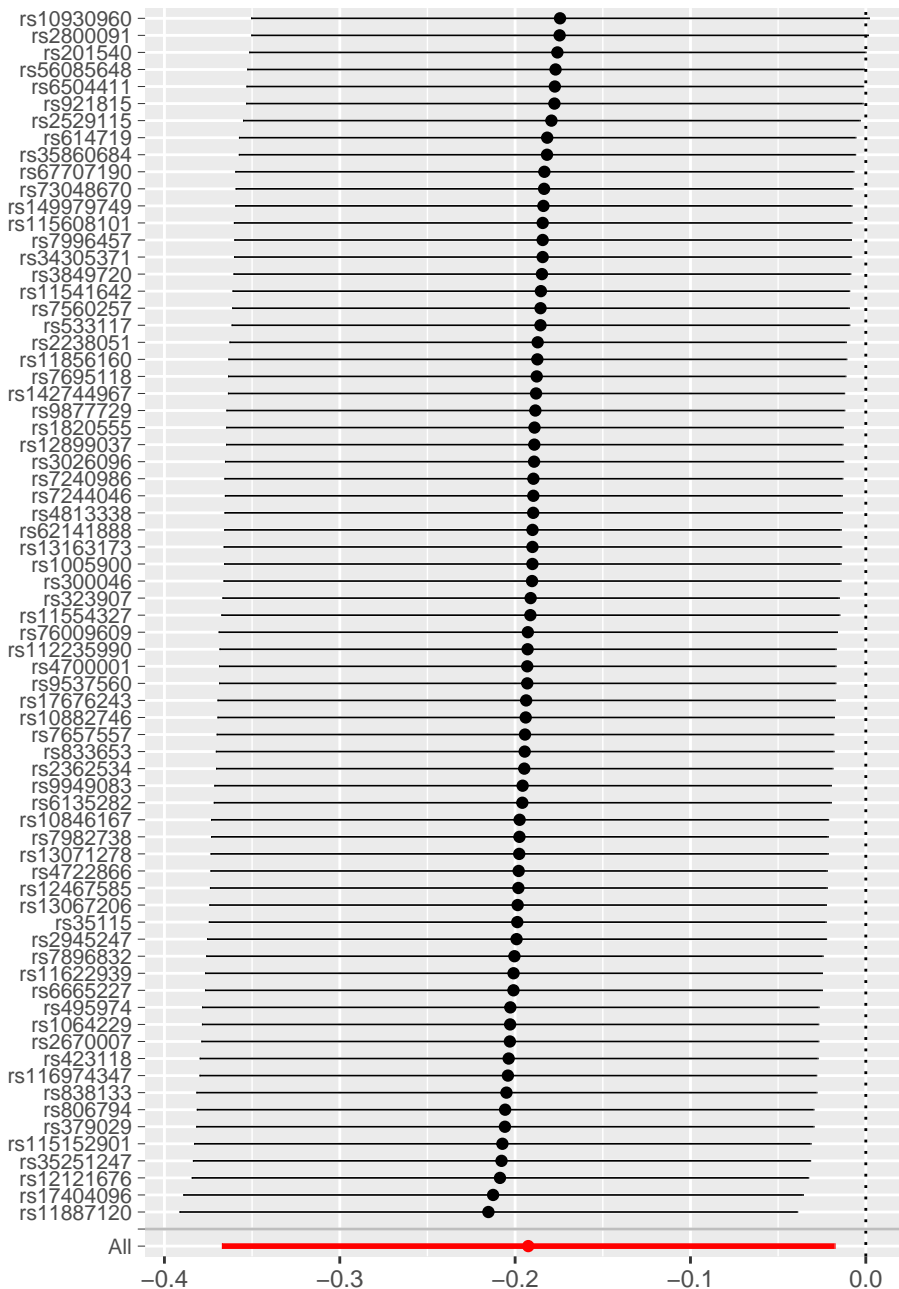

Supplement: Supplementary file 1 [file nutrients-16-02433-s001.zip › Supplementary materials/Figure S6 Leave-one-out plot of MR analysis between processed meat intake and telonere length.pdf]
